# Localization of Integrin Beta-4 Subunit at Soft Tissue–Titanium or Zirconia Interface

**DOI:** 10.3390/jcm9103331

**Published:** 2020-10-17

**Authors:** Yasunori Ayukawa, Ikiru Atsuta, Yasuko Moriyama, Yohei Jinno, Kiyoshi Koyano

**Affiliations:** 1 Section of Implant and Rehabilitative Dentistry, Division of Oral Rehabilitation, Faculty of Dental Science, Kyushu University, 3-1-1 Maidashi, Higashi-ku, Fukuoka 812-8582, Japan; kabay@dent.kyushu-u.ac.jp (Y.M.); jinno315@dent.kyushu-u.ac.jp (Y.J.); koyano@dent.kyushu-u.ac.jp (K.K.); 2 Division of Advanced Dental Devices and Therapeutics, Faculty of Dental Science, Kyushu University, 3-1-1 Maidashi, Higashi-ku, Fukuoka 812-8582, Japan; atyuta@dent.kyushu-u.ac.jp

**Keywords:** dental implant, titanium, zirconia, integrin α6β4, peri-implant epithelium

## Abstract

Currently, along with titanium (Ti), zirconia is widely used as an abutment material for dental implants because it makes it possible to avoid gingival discoloration; however, the epithelial sealing capability of zirconia remains unknown. The purpose of the present study is to elucidate the localization of integrin β4 subunit (Inβ4), one of the main proteins in the attachment structure between gingival junctional epithelial (JE) cells and substrata. Maxillary first molars were extracted from rats, and implants were placed with Ti or zirconia transgingival parts; then, the localization of Inβ4 was observed. Morphological and functional changes in rat oral epithelial cells (OECs) cultured on a culture dish (Dish) and Ti and zirconia plates were also evaluated with Inβ4 immunofluorescence histochemistry and Western blotting. After four weeks of implant placement, the morphology of the peri-implant epithelium (PIE) and the localization of Inβ4 around the Ti and zirconia transgingival parts were similar. However, both exhibited markedly shorter Inβ4-positive bands in the PIE than in the JE around natural teeth. Decreased expression levels of Inβ4 were observed in OECs cultured on Ti and zirconia plates compared with those cultured on Dish. In conclusion, although inferior to natural teeth, zirconia implants are thought to have epithelial sealing properties comparable to those of titanium.

## 1. Introduction

Possessing excellent characteristics for osseointegration and soft tissue integration, titanium (Ti) is widely used as a dental implant material. However, its metallic color can occasionally be seen through the gingiva, and this gingival discoloration decreases soft tissue esthetics. In patient-defined success criteria for dental implant treatment, esthetics and occlusal function are emphasized. In this context, zirconia was introduced in implantology owing to its superb physical strength and white color, avoiding gingival discoloration [1,2].

In the case of natural teeth, a strong sealing structure termed “biologic width” is formed at the tooth–gingiva interface. Specifically, the junctional epithelium (JE) strongly attaches to the enamel surface with hemidesmosomes. Integrin (In) α6β4 is a transmembrane protein dimer of JE cells, and it is a main component of hemidesmosomes [3,4]. This protein dimer operates as a receptor of laminin-332, which is a major component of the basal lamina on the enamel surface [5]. This anchorage between Inα6β4 and laminin-332 offers a secure seal between enamel and JE.

The cervical portion of the implant component can be a source of inflammation around an implant because the implant penetrates the oral mucosa, and plaque control in this area is often insufficient. Although peri-Ti implant soft tissue was reported to possess biologic width and hemidesmosome–basal lamina connection, its sealing capability is inferior to that of periodontal biologic width [6,7]. By using zirconia as a material for the transgingival part of the implant, equal or improved soft tissue attachment can be achieved compared with that of Ti.

We therefore focused on the localization of integrin β4 subunit (Inβ4) at the interface between gingival soft tissue and zirconia. As mentioned above, Inβ4 is a hemidesmosome constituent, and can be an indicator for the secure attachment of PIE to zirconia. In the present study, the immunohistochemical localization of Inβ4 was investigated, and the distribution of Inβ4 with that around Ti and teeth was assessed using animal model and cell culture studies.

## 2. Materials and Methods

### 2.1. Ethical Approval

All experiments were performed in accordance with the ARRIVE Guidelines for reporting animal research [8]. All procedures involving experiment animals were approved by the Institutional Animal Care and Use Committee of Kyushu University (approval number: animal implantation experiment: A25-240-0; cell culture experiment A21-237-0).

### 2.2. Experimental Implants

**Figure 1:**
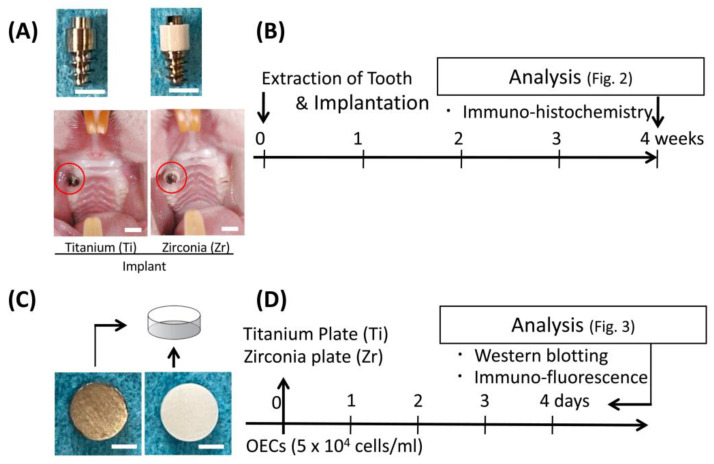
Implants and plates used in the present study and experimental design. (**A**) Photographs of implants with titanium or zirconia transgingival insert (upper panels), and intraoral view of rat after implant placement (lower panels); bar = 2 mm. (**B**) Experiment protocol for in vivo study. Implantation performed immediately after tooth extraction. (**C**) Photographs of titanium and zirconia plates; bar = 5 mm. (**D**) Experiment protocol of in vitro study.

The detailed characteristics of the implants used in the present study were previously reported [9]. In brief, two-piece implants, consisting of a Ti (Grade 2 ASTM F67) screw as an intrabony portion and a transgingival insert made of Ti (Grade 2 ASTM F67) or zirconia (0.05 wt% Al_2_O_3_-doped 3.0 mol% Y_2_O_3_-stabilized tetragonal zirconia polycrystal, Tosoh, Tokyo, Japan), were fabricated (Sky Blue, Fukuoka, Japan). The dimension of the transgingival column was 4 mm in height and 2 mm in diameter (Figure 1A). The Ti screw and transgingival part were engaged with each other using friction. The surface roughness value (Ra) of the transgingival part of each implant was 0.86 ± 0.033 μm in Ti and 0.13 ± 0.025 μm in zirconia [10].

### 2.3. Implantation

The experiment protocol was previously reported [11]. In brief, 6-week-old Wistar rats (20 males; 120–150 g) underwent extraction of maxillary right first molars under systemic anesthesia. Immediately after extraction, the extraction socket was enlarged using a dental reamer (80 to 120, Torpan, Maillefer, Switzerland), and the experimental implant was then screwed into the socket (Figure 1A,B).

### 2.4. Immunohistochemistry for Animal Experiments

At 4 weeks after implantation, all rats were euthanized. The oral mucosa was removed from the maxillary bone, and sections were cut on the coronal plane using a cryostat (−20 °C) after demineralization using a 5% ethylenediaminetetraacetate solution. For immunohistochemical staining, these sections were incubated with rabbit anti-Inβ4 (1:100 dilution, Chemicon International, Billerica, MA, USA) and biotinylated anti-rabbit IgG (1:100 dilution, Sigma-Aldrich, St. Louis, MO, USA), and the presence of Inβ4 was visualized using a diaminobenzidine (DAB) staining kit (Vector Laboratories, Burlingame, CA, USA), as described in a previous paper [11]. In the present study, the vertical distance between the top of the PIE or JE and the bottom of the Inβ4-positive area was measured in a direction parallel to the implant or natural tooth (Nt) surface.

### 2.5. Statistical Analysis

Data are indicated as means ± standard deviation (SD). One-way analysis of variance with post hoc Scheffe’s test was performed. Values of *p* < 0.05 were considered statistically significant.

### 2.6. Culture Experiments

Zirconia (0.05 wt% Al_2_O_3_-doped 3.0 mol% Y_2_O_3_-stabilized tetragonal zirconia polycrystal) and pure Ti (Japan Industrial Specification Class 1, H 4600, 99.9 mass%) plates (15 mm diameter, 1 mm thickness, Sky Blue) were used for this experiment. The surface roughness value (Ra) of each plate was identical to the implant surface for the animal study. Oral epithelial cells (OECs) were obtained from the oral mucosa of 4-day-old Wistar rats as reported previously [11,12,13]. Briefly, oral mucosa were incubated with dispase (1 × 10^3^ IU/mL) in Mg^2+^- and Ca^2+^-free Dulbecco’s phosphate-buffered saline for 12 h at 4 °C and removed from the connective tissue layer. Cells were cultured in Defined Keratinocyte Serum Free Medium (DK-SFM; Thermo Fisher Scientific, Waltham, MA, USA) to eliminate contamination with cells other than epithelial on a culture dish (Dish; Thermo Fisher Scientific) and Ti and zirconia plates (Figure 1D) at 37 °C in a humidified atmosphere of 5% CO_2_ in air.

### 2.7. Immunofluorescence Staining for Culture Experiment

After 4 days of culture, OECs were fixed with 4% paraformaldehyde. Samples were then incubated with a polyclonal mouse anti-Inβ4 antibody (1:100 dilution, Santa Cruz Biotechnology, Dallas, TX, USA) and fluorescein isothiocyanate-labeled secondary antibody (1:100 dilution; Chemicon International). Actin filaments were stained with tetramethylrhodamine isothiocyanate-conjugated phalloidin (1:100 dilution, Sigma-Aldrich, St. Louis, MI, USA). Imaging was performed using fluorescence microscopy (BZ-9000; Keyence, Osaka, Japan).

### 2.8. Western Blotting

Proteins were separated by sodium dodecyl sulfate-polyacrylamide gel (7.5%) electrophoresis, transferred to polyvinylidene difluoride membranes (Bio-Rad Laboratories, Hercules, CA, USA) and immunoblotted with the anti-Inβ4 antibody. Antibody-bound bands were visualized using an imaging device (LAS500, GE Healthcare, Chicago, IL, USA).

## 3. Results

### 3.1. In Vivo Localization of Inβ4 around Experimental Implant

**Figure 2:**
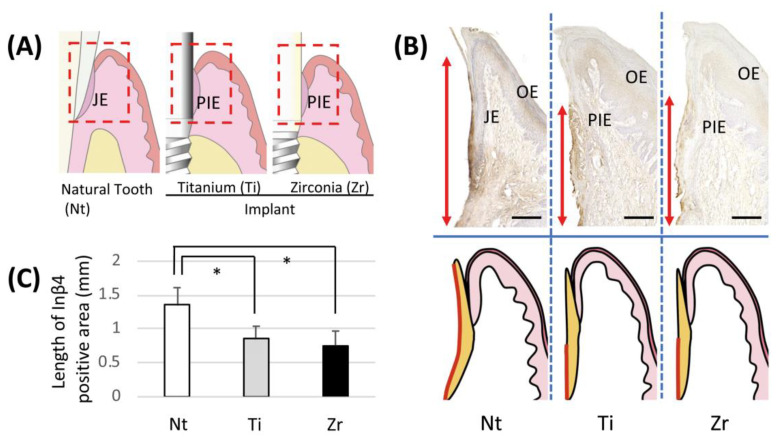
Immunohistochemical localization of integrin β4 (Inβ4) in junctional epithelium (JE) and peri-implant epithelium (PIE). (**A**) Schemes of periodontal and peri-implant structures. Dotted red line indicates observed area. (**B**) Immunohistochemical localization of Inβ4 in JE or PIE around natural tooth (Nt) or implants with titanium (Ti) or zirconia (Zr) transgingival insert. Schematic diagrams of soft tissue structures around teeth or implants shown in lower panels. Red arrows (upper panel) and lines (lower panel) indicate range of Inβ4-positive area; bar = 200 μm. (**C**) Mean Inβ4 subunit-positive lengths on JE or PIE around Nt, Ti and Zr. Data represent means ± SD of three parallel experiments. * *p* < 0.05.

A positive band of Inβ4 was observed at JE along the surface of the enamel (Figure 2B, left). In both implant groups, however, the Inβ4-positive band was limited to the apical portion compared with the Nt group. The expression pattern of Inβ4 on the zirconia surface was similar to that on Ti (Figure 2B, center and right). In addition, the lengths of the positive staining bands in both implant groups were significantly shorter than that in the Nt group (Figure 2C).

### 3.2. In Vitro Localization of Inβ4 in OECs on Titanium or Zirconia Plate

**Figure 3:**
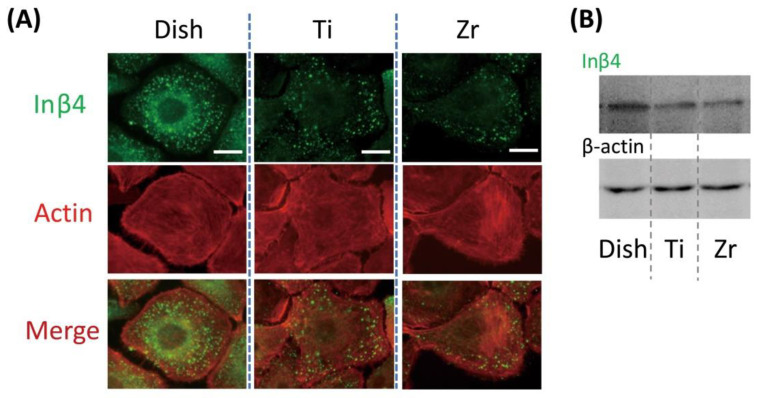
Expression of Inβ4 in oral epithelial cells (OECs) on culture dish (Dish), titanium (Ti) and zirconia (Zr) plates. (**A**) Expression of Inβ4 subunits and development of actin filaments in OECs in vitro; bar = 15 μm. (**B**) Western blot analyses of Inβ4.

The signal of Inβ4 was scattered in the cytoplasm of OECs seeded on Ti and zirconia plates (Figure 3A, center and right), while reactions in OECs on the Dish were observed in the cytoplasm and around the nucleus (Figure 3A, left). The number of positive Inβ4 reactions on the Ti and zirconia plates appeared to be lower than that on the Dish (Figure 3A, Row 1). OECs on the Ti and zirconia plates had an actin filament with more interruptions than that on the Dish (Figure 3A, Row 2). Western blot analysis presented similar findings to those of immunofluorescence results, that is, OECs on Ti and zirconia plates had slightly weaker expressions of Inβ4 than those on the Dish (Figure 3B).

## 4. Discussion

Peri-implantitis and subsequent tissue breakdown are the main reasons for the loss of implants [14]. Weakness in the attachment between titanium implants and the surrounding soft tissue was reported, and this weakness is ascribed to insufficient amounts of attachment structures such as hemidesmosomes and basal laminae [7,15]. The attachment structure between the gingival epithelium and substrata such as enamel or implants comprises two components, namely basal lamina and cell-adhesion protein. Previous studies indicated that the length of the band of laminin-332, which is a main component of basal lamina, is shorter around Ti implants than around the enamel [7,11]. In contrast, another component of the attachment structure, transmembrane receptors in epithelial cells, has not yet been studied in detail. As indicated above, laminin-332 is a main component of the basal lamina between Ti and epithelial cells, the localization of Inα6β4, which is known to be a receptor of laminin-332 [16], may further elucidate the nature of attachment between implants and epithelial cells. In the present study, the localization of Inβ4 between the implant material and epithelium was studied using cell culture and animal studies. Zirconia is a relatively new material for use in dental implants, and the attitude of epithelial tissue with respect to zirconia is still to be clarified. We therefore studied the localization of Inβ4 around zirconia and Ti, and compared the epithelial attachment to both materials.

In our animal study, there was no statistically significant difference in the length of the Inβ4-positive band between Ti and zirconia groups, both of which were significantly shorter than that for teeth. As previously reported, the length of the laminin-332-postive band on the substratum side of the attachment structure was significantly shorter around Ti than that around teeth [7,11]. The present study showed that the localization of Inβ4 on the cell side of the attachment structure was similar to that of laminin-332. In addition, it could be speculated that the sealing capability around Ti and zirconia was similar because Inβ4 exhibited a similar localization pattern around both materials.

Our cell culture study indicated that the expression of Inβ4 in gingival epithelial cells was also similar between Ti and zirconia groups, and they were significantly inferior to those seeded on the culture dish. Specifically, in cells cultured on the culture dish, Inβ4 signals were observed around nuclei and in the cytoplasm. This means that Inβ4 was synthesized in cells cultured on the culture dish. In contrast, Inβ4 was not actively synthesized in cells seeded onto Ti or zirconia. Western blot analysis also showed a similar tendency. According to a previous report, the expression of Inβ4 on the Ti plate is weaker than that on the culture dish [11], and this is consistent with the present study. Similar expressions of Inβ4 in cells cultured on Ti and zirconia were also consistent with our animal study. Another previous report indicated that osteoblastic cells seeded onto hydrophilic surfaces presented a superior cell attachment count in comparison to those seeded onto hydrophobic titanium surfaces [17], and both αv and β3 integrin subunits were downregulated in osteoblastic cells on hydrophobic surfaces [18]. Although the cell and subunits of integrin were different from those used in the present study, results are consistent with our own. In addition, our recent study indicated that the generation of a hydrophilic surface on titanium using hydrothermal treatment enhanced the adsorption of laminin-332 [19]. It can be speculated that the addition of hydrophilicity to Ti and zirconia enhances the expression of Inβ4 through the promotion of laminin-332 adsorption onto substrata and the subsequent strengthening of epithelial attachment.

The arrangement of actin fibers was also inferior in cells cultured on both materials. This also implies that the attachment of epithelial cells to both Ti and zirconia is weaker than that to the culture dish.

The limitation of the present study is that rats were used as an in vivo oral implantation model. Although this model has been widely used [6,7,11,15,20], the monitoring of occlusion, and any other force and plaque control measures, is impossible, and these factors may influence soft tissue attachment. In addition, while the localization of Inβ4 may allow for us to estimate the secureness of epithelial attachment, the actual sealing capability of epithelial attachment should be further studied.

On the basis of our results, zirconia can also be used as a material for the transgingival part of implants from the point of view of epithelial attachment. Since this material can avoid gingival discoloration, further knowledge such as on mechanical properties and plaque retentivity should be accumulated, with the aim of using this material for the transgingival part of implants with excellent long-term prognosis.

## 5. Conclusions

Within the limitation of the present study, we conclude that the expressions of Inβ4 in epithelial cells on Ti and zirconia were similar and inferior to those in periodontal epithelial cells. This implies that, from the viewpoint of epithelial attachment, although it is obvious that both materials are inferior to enamel, zirconia can be used as the transgingival part of an implant in an equivalent manner to that of Ti.
